# Humoral Immunity against Measles in Mother–Infant Pairs during the First Year of Life in Greece: A Cross-Sectional Study

**DOI:** 10.3390/vaccines9020143

**Published:** 2021-02-10

**Authors:** Florentia Kanakoudi-Tsakalidou, Evangelia Farmaki, Eleni Papadimitriou, Anna Taparkou, Eleni Agakidou, Styliani Glykou, Fotiοs Papachristou

**Affiliations:** 1st Department of Pediatrics, Faculty of Medicine, Aristotle University of Thessaloniki, Ippokrateion General Hospital, 49 Konstantinoupoleos Str, P.C. 54246 Thessaloniki, Greece; farmakg@med.auth.gr (E.F.); epapadimitriu@gmail.com (E.P.); annatapark@gmail.com (A.T.); eagaki@hotmail.com (E.A.); stella83g@gmail.com (S.G.); fotios@med.auth.gr (F.P.)

**Keywords:** maternal-infant measles immunity, measles antibodies, waning measles immunity, vaccine-induced immunity, measles susceptible infants, measles passive immunity

## Abstract

Measles outbreaks have surfaced in Europe during the last decades. Infants <12 months of age were the most severely affected pediatric population. The aim of this study was to investigate the duration of maternally derived measles antibodies in infants aged 1 to 12 months in relation to maternal humoral immune status and other parameters. In a prospective, cross-sectional cohort study, 124 mother/infant pairs and 63 additional infants were recruited from October 2015 through December 2019. Infants were hospitalized in a university pediatric department of a general hospital. Demographic and epidemiological data were recorded and blood samples were collected from mothers and their infants. Commercially available enzyme-linked immunosorbent assay (ELISA) was used for measuring measles antibodies. Fifty nine percent of mothers had vaccine-induced and 15% infection-acquired measles immunity. Eighty-eight percent and 94% of infants were unprotected by 5 and 10 months of age, respectively. Maternal antibody levels and infant age were significant independent predictors of infants’ antibody levels whereas the method of maternal immunity acquisition, age, and origin [Greek/non-Greek] were not. Our findings suggest that about 90% of infants are susceptible to measles beyond the age of 4 months. To our knowledge, these are the first data from Greece reported under the current community composition and epidemiological conditions.

## 1. Introduction

Measles and rubella had been targeted for eradication in Greece by 2020, as in the whole World Health Organisation (WHO) European Region, following the WHO Global Measles and Rubella strategic plan 2012–2020 [1]. After 2011, only sporadic measles cases were recorded in the country. Thereafter, no endemic transmission was identified for a period of 36 months in 2014–2016 and according to the European Regional Verification Commission for Measles and Rubella elimination, Greece was officially recognized as having achieved elimination [2]. The “measles elimination settings” are defined as lack of endemic transmission of the virus for ≥12 months [3]. In these settings, most women of childbearing age have obtained their immunity following immunization rather than by a community-acquired measles infection and therefore their measles immunoglobulin gamma (IgG) levels are gradually waning with time, without a chance for immune boosting. Thus, sporadic measles outbreaks could occur from year to year, presumably due to imported cases, potentially causing severe complications and deaths [4,5,6]. The proportion of affected infants in some outbreaks may reach 31% [7]. Previous studies have shown that infants born to measles-vaccinated mothers have lower levels of maternally derived antibodies at birth and a shorter duration of protection than infants born to measles-infected mothers [8,9,10,11,12,13,14,15,16].

In most measles elimination settings, infants receive the first dose of vaccine against measles, mumps, and rubella (MMR) around the 12th month of age (12–15th month in Greece). Consequently, a gap of susceptibility emerges between the age at which infants lose their maternal antibodies and the age of vaccination implementation [13]. Therefore, it is important to reduce this period of susceptibility because the risk of measles severity is greater in infants aged <12 months [4]. On the other hand, determining the optimal timing for the first vaccine dose has a vital role in keeping this period as short as possible [13].

During the last two decades, the massive influx of refugees and immigrants in Greece, as in many other countries, resulted in altering both population composition and epidemiological conditions. In 2015 the Hellenic Centre for Disease Control and Prevention (HCDCP) informed the Greek physicians about the risk of an upcoming measles outbreak and urged them to increase the vaccination rate with MMR, targeting vaccine coverage of ≥95%. According to recently published data [17], to eliminate measles in Europe, it is necessary to succeed percentages of complete vaccination coverage of at least 97% and measles immunity levels in children aged 1−9 years of at least 95%. In Greece, the percentage of one dose vaccine coverage with MMR in preschool children aged 2–3 years is deemed satisfactory, reaching 97.3% [18]. However, infants continue to be susceptible to measles for an extended period prior to their first vaccine dose.

Following the warning of the HCDCP, we felt that it would be important to determine the immune status against measles in infants throughout the first year of age, as current relevant data from Greece were lacking at the time. To this aim, we assessed the antibody levels against measles in pairs of full-term infants aged 1–12 months and their mothers who are residents of Greece. In addition, we investigated the relation of infant antibody levels with certain maternal parameters. The results of such a study could possibly contribute to a re-evaluation of the current public health policy concerning the age for commencing and completing vaccination against measles.

## 2. Study Population and Methods

Participants eligible for inclusion in this prospective, cross-sectional, cohort study were healthy mothers and their full-term infants (≥37 weeks gestational age) aged 1–12 months (5–51weeks) who were hospitalized in a University Pediatric Department, at a General Hospital, from October 2015 to December 2019. The reasons for hospitalization were acute respiratory or urinary tract or gastrointestinal infections, according to the inclusion criteria (Table 1). Exclusion criteria are also synopsized in Table 1. The duration of hospitalization ranged from 4 to 8 days. All parents gave informed, written consent for their children’s participation prior to their inclusion in the study.

The study protocol was approved by the Ethics Committee of Ippokrateion General Hospital of Thessaloniki (protocol number 280/8-04-2015) and all work was conducted in accordance with the declaration of Helsinki of 1975 [available on line: https://www.wma.net/what-we-do/medical-ethics/declaration-of-helsinki/ (accessed on 18 August 2020)], revised in 2013. All mothers completed a questionnaire developed by the authors for the needs of the study, including history of measles infection or vaccination plus demographic, clinical, and epidemiological data.

Blood samples were collected concomitantly with blood sampling for routine tests and centrifuged at 2000× *g* for 10 min within 30 min following collection. Supernatant serum was removed and aliquots were stored at −70 °C until tested. All samples were tested in duplicates.

Commercially available enzyme-linked immunosorbent assay (ELISA) was used for detection of IgG antibodies against measles virus, according to the manufacturer’s instructions (Virion/Serion, Würzburg, Germany). “SERION ELISA classic Measles Virus IgG” permits detection of antibody activity in international units [IU]. The quantitative results were measured in mIU/mL according to the manufacturer’s instructions. Interpretation of the results was as follows: concentrations > 200 mIU/mL were considered as positive, 150–200 as equivocal [intermediate], and <150 as negative for humoral immunity against measles.

## 3. Statistical Analysis

Continuous variables were expressed as either means and standard deviations or medians and interquartile range (IQR) depending on value distribution (Kolmogorov–Smirnov test). Categorical variables were expressed as counts and percentages. Comparisons between/among groups were performed using the Mann–Whitney U test, the Kruskal–Wallis analysis of variance (ANOVA), and the Fischer’s exact test, as appropriate. Bivariate correlations were assessed using the Spearman correlation coefficient. A multiple regression analysis model was constructed with dependent variable the infants’ humoral immune status, while factors known to be related with the infants’ humoral immune status and those differing significantly on comparisons between/among subgroups were included in the model as independent variables. Analysis was performed using the generalized linear models—ordinal logistic response, as the dependent variable was a multi-class ordered variable, i.e., categorical variable following an order (e.g., negative, equivocal, positive). A two-sided *p* value of <0.05 was indicative of statistical significance, while Bonferroni adjustment was applied for multiple comparisons. Statistical analysis was performed using the IBM SPSS Version 23 (IBM Co., New York, NY, USA).

## 4. Results

### 4.1. Demographic and Epidemiological Data

During the study period, 187 full-term infants with age varying between one and 12 months and their healthy mothers met the inclusion criteria. Demographic and epidemiological data of mothers and infants are shown in Table 2. Median infants’ age at recruitment was 8 months and 60% of them were males. Maternal history of measles infection or vaccination against measles was identified in 74.3% of mothers whereas 25.7% of them could not recall and were registered as having unknown “measles history”. Comparison of demographic, epidemiological, and laboratory data between Greek and non-Greek mothers are shown in Table 2. The national origin of the non-Greek mothers was Albania (28/51), Russia, Georgia, Italy, Germany, Poland, Bulgaria, Rumania, Ukraine, and Armenia. The non-Greek mothers were residents in Greece since a median age of 15 years (range between 3 and 25 years). All infants studied were born and live in Greece.

One hundred and twenty-four mothers (66.3%) consented to be tested along with their infants for detection of measles antibodies and 63 (33.6%) consented only to their infants’ blood testing. Recruitment was extended until December 2019 due to difficulties in obtaining parental written consent for infants less than 6 months of age. In the meantime, an outbreak of measles occurred in Greece (May 2017–October 2018) with 3298 cases (11% or 363 aged <1 year and 25.4% aged 1–4 years) [2]. Publicity of the epidemic data influenced the parent’s attitudes, so that we were able to recruit additional infants less than six months of age. No mother or infant included in our study developed measles over the study period. In total, 311 blood samples were collected from 124 mother/infant pairs and 63 additional infants. The infants were subdivided into four age-related subgroups; subgroup 1 aged 1–4 months (5–16 weeks), subgroup 2 aged 5–7 months (17–29 weeks), subgroup 3 aged 8–9 months (30–40 weeks), and subgroup 4 aged 10–12 months (41–51 weeks, Table 3). Maternal age, history of measles infection or vaccination, and measles antibody levels did not differ significantly among the four infant age-related subgroups.

### 4.2. Humoral Immunity against Measles in Mothers and Infants

**Figure 1:**
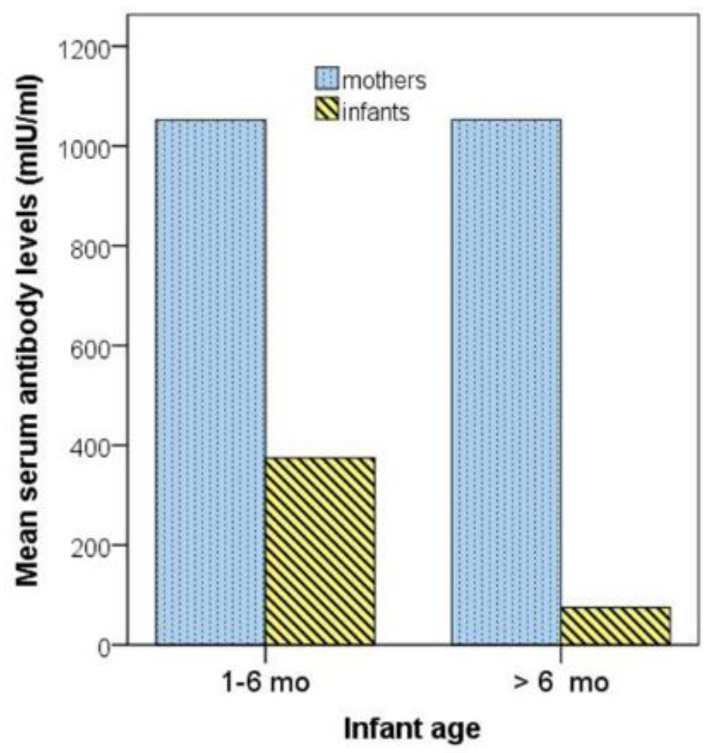
Serum measles antibody levels in two infant subgroups with age 1–6 months and >6 months, respectively, and their respective mothers. mo, months.

Serum measles antibody levels in two infant subgroups with age lower than 6 months and equal to/higher than 6 months, respectively, and their respective mothers are depicted in Figure 1.

**Figure 2:**
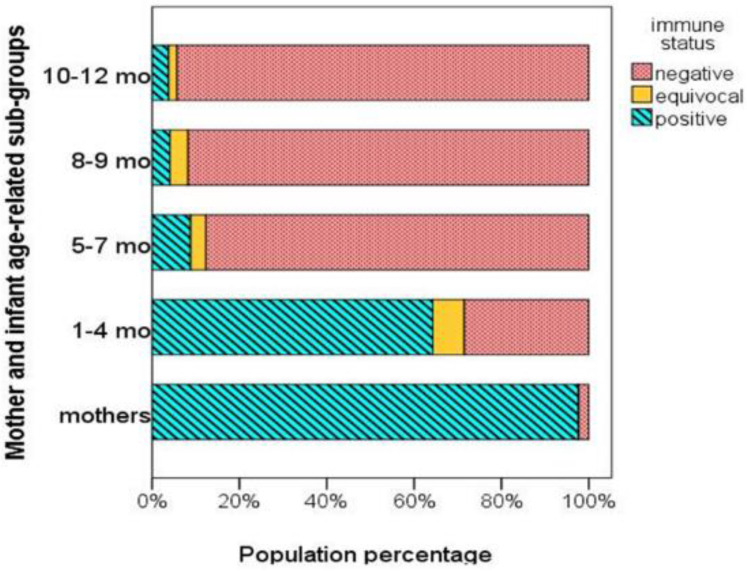
Percentages of mothers and infants of different age-related subgroups with protective (positive), equivocal, and non-protective (negative) measles antibody levels.

In addition, the proportion of mothers and infants of the four age-related subgroups with antibody levels above or below the protective threshold (>200 mIU/mL or <150 mIU/mL, respectively) are depicted in Figure 2. Both Figure 2 and Table 2 show that measles antibody levels were above the protective threshold in the vast majority of mothers (97.6%), while being below the protective threshold in the majority of infants (81.8%). Mothers with a history of measles infection had higher antibody levels than mothers with vaccine-induced immunity (median [IQR], 1322 [923] mIU/mL vs. 1086 [876] mIU/mL), but the difference was not significant (*p* = 0.158). Moreover, there was no correlation between maternal antibody levels and maternal age (r = 0.052, *p* = 0.576).

The infants’ antibody levels did not differ significantly between infants born to mothers with infection-acquired immunity [median = 55.7 and IQR = 10.9–1402] and those born to mothers with vaccine-induced immunity (median = 10.6 and IQR = 106.9–1402, *p* = 0.576). No difference in infants’ antibody levels was found between boys and girls (median [IQR], 51.7 [104] and 65.0 [106], for boys and girls, respectively, *p* = 0.628) as well as between infants born to mothers of Greek and non-Greek national origin (*p* = 0.349, Table 2).

### 4.3. Infant Measles Passive Immunity in Relation to Infant Age

**Figure 3:**
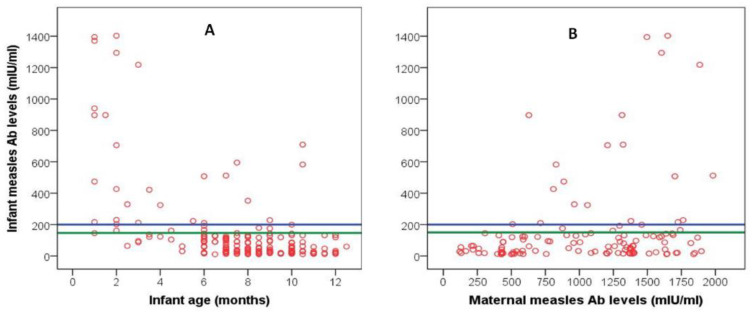
Scattergram of infant measles antibody levels plotted against infants’ age (**A**) and maternal antibody levels (**B**). The blue reference line represents the lower protective level (200 mIU/mL); the area between the blue and green reference lines represents the area of equivocal protective levels (150–200 mIU/mL).

The distribution of infants’ measles antibody levels plotted against infants’ age and mothers’ antibody levels is shown in Figure 3. The median values of measles antibody levels in the four age-related subgroups are shown in Table 3. Comparison among groups showed that a significantly lower percentage of the subgroup 1 (1–4 months) had unprotected antibody levels (<150 mIU/mL) compared to the other three age-related subgroups (*p* < 0.001). After the 4th month, the infants’ passively acquired measles immunity waned significantly so that between the 5th and 12th month ≥89% of infants were susceptible to the disease. On bivariate correlations, infants’ antibody levels were negatively correlated with infants’ age (r = −0.510, *p* < 0.001, Figure 3A) and positively with maternal antibody levels (r = 0.220, *p* < 0.014) (Figure 3B), while they were not correlated with maternal age.

### 4.4. Multiple Regression Analysis

A model of multiple regression analysis was performed to investigate the potential independent association of infants’ humoral immune status with infant-age subgroups, maternal measles antibody concentrations, national origin, history of measles infection or vaccination, and age. Analysis confirmed that the infant age-related subgroups (specifically subgroup 1) and maternal antibody levels were significant independent predictors of the infant’s immune status after adjusting for confounders.

## 5. Discussion

The findings of our study showed that most infants in our cohort were born to mothers who obtained their measles immunity following immunization (58.8%) rather than through infection (15.5%). It was also evident that maternally derived antibodies waned significantly after the 4th month of age. Specifically, 87.7% of infants were found to be unprotected around the age of 5–7th month, while the proportion increased with age reaching 94.3% by the 10–12th month.

Among the various maternal parameters studied, we revealed that maternal antibody levels significantly correlated with the infants’ antibody concentrations before and after adjustment for potential confounders. The maternal age in our study did not correlate with the infants’ antibody titers. In fact, although Greek mothers were significantly older than the mothers of other national origin, the mean measles antibody concentrations were significantly higher in the former than in the latter group (*p* = 0.002). A similar finding is also discussed by Science et al. [7] in a recent relevant study. The authors had no data of immigrant mothers’ country of birth or any vaccination history recorded and despite the fact that many of them may have originated from countries where measles is still endemic, their antibody levels were low [7]. In our cohort, no difference was found between Greek and immigrant mothers regarding the way they had obtained their measles immunity. Considering that 23% and 31% of the Greek and immigrant mothers, respectively, had unknown measles infection/vaccination history, we cannot evaluate this finding definitively.

Our findings concerning the early loss of maternally derived antibodies in infants, resulting in a long interval of susceptibility between antibody waning and infant immunization, are in agreement with studies conducted either in elimination settings like Greece, or in low incidence, non-elimination ones [8,9,10,11,12,13,14,15,16]. However, our results do not confirm findings of studies showing that measles antibody levels in infants born to mothers with vaccine-induced immunity are lower compared to that of infants born to mothers with infection-acquired immunity [8,9,10,11]. This difference could be attributed to the very low proportion of mothers with infection-acquired immunity in our study population.

Our findings regarding the duration of maternally-derived immunity are consistent with those reported previously [7,13,14,15,16]. Waainborg et al. [13], in a large cross-sectional serologic survey in Netherlands communities with contrasting vaccination coverage, estimated that the duration of protective passive immunity among infants born to mostly vaccinated mothers was 3.3 months. The authors outline the fact that in measles elimination settings with highly vaccinated populations, infants lose protection at an earlier age (two months earlier) than infants of mothers who live in communities that oppose vaccinations in general and hence have lower vaccine coverage. Boulton et al. [14], in their study on infant/mother pairs from a sample of immunization clinics in all Tianjin districts, China, found that almost all infants lost protection by 3 months regardless of the antibody titer in their mothers. They comment that many cases of measles in Tianjin occur in infants whose mothers were born following implementation of widespread vaccination programs.

Science et al. [7] studied susceptibility to measles over the first year of life in a large cohort of infants, in Ontario Canada (an elimination setting). They found that the majority of infants are susceptible to measles by 3 months of age, well before immunization with the first dose of measles vaccine, administered at 12 months. They outline that despite Canada’s elimination status, there are still sporadic measles outbreaks annually due to imported cases from abroad with the proportion of affected infants consisting the 31% of measles cases in some years [7]. In another, single-center, prospective study from Spain, Cilleruello et al. [16] measured longitudinally measles, rubella, and mumps antibodies in 146 mother-child pairs at 3, 6, 9, and 12 months after childbirth. The titer of measles antibodies in infants declined after the 3rd month, while no antibodies were detected by the 6th month. They suggest that the MMR vaccination could commence at the age of 9 months provided that larger population studies confirm their results.

Although different methods for measuring measles antibodies have been used in the aforementioned studies (mainly ELISA and the plaque reduction neutralization test, PRNT), results are similar and their data lead to a common conclusion; in settings of sustained measles elimination, most infants are vulnerable to measles well before the age of routine measles immunization. In order to keep this vulnerability period as short as possible, we have to define the optimal timing for the first vaccine dose, on the basis of setting-specific seroprevalence in various eras [15]. This is not an easy task, considering that a successful immunization depends mainly on the infant’s immune system capability to induce an efficient immune response. Moreover, levels of maternal antibodies must be low enough so that the attenuated vaccine strain will not be neutralized. Thus, we have to achieve a balance between the risk of infection and these assumptions. Several studies in the past decades have tried to identify the appropriate age for starting measles vaccination, recommending different ages and dose schedules between 6 and 15 months [19,20,21,22,23,24]. Results of these studies are not always consistent. For example, Gans et al. in a study of passive and active immunity during the first year of life supported that protective immunity against the measles virus depends on both humoral and cellular immune response [21,22]. The authors propose two-dose vaccination schedule; the first dose at the age of 6 months, in order to induce the “priming” of the immune system regardless of the strength of the immune response and the second at the 12–15th month of age to achieve a long-lasting protective immunity. Some researchers have also come to the same conclusions [20], whereas others propose the age of 15 months as more appropriate to achieve a better and long-lasting immune response [23]. In our opinion, this ongoing debate is no longer necessary, as the “two–dose schedule” of vaccine administration has been adopted by many countries worldwide. Using this schedule, both the priming (by the fist dose) and long-lasting immune memory (by the second dose) are achieved, resulting in long-lasting protection. However, the point is not the need of two doses for measles vaccine but rather when to start vaccination safely and efficiently. In this sense, most relative publications from different countries worldwide, including ours, highlight that there is an extended period of susceptibility to measles in infancy and recommend administering the first vaccine dose at the age of nine months [13,14,15,16].

A limitation of our study could be the recruitment of different infant cohorts in each age-subgroup instead of assessing longitudinally the measles antibody levels in the same infant cohort which, however, would present ethical issues and would reduce the total number of infants studied overtime [16]. The single center design could be considered as another limitation. However, our pediatric department accepts referrals of infants and children from all over Northern Greece. The strength of our study is the prospective assessment of humoral immune status in a considerable number of mother–infant pairs and the collection of maternal demographic and epidemiological data using a pre-scheduled questionnaire.

## 6. Conclusions

It is obvious that measles epidemiology and immunity have changed, particularly in elimination or eliminating settings with high vaccination coverage where outbreaks of the disease continue to occur. Data derived from recently published studies from different continents, including the current study, lead to similar conclusions concerning infants’ vulnerability to measles; mothers who are immune against measles may be unable to protect their children beyond the sixth month by means of passively transferred immunity. We believe it is time for the expert committees around the world to re-evaluate and tailor vaccination programs against measles [25,26] to countries’ current epidemiological conditions.

## Figures and Tables

**Table 1:** Inclusion and exclusion criteria.

1. Inclusion criteria
Full term infants
Age >1 month to 12 months (5 to 51 weeks)
Acute respiratory or gastrointestinal infections not affecting the general condition
Urinary tract infection
2. Exclusion criteria
Severe acute illness
Chronic diseases
Underlying diseases associated with immunoglobulin depletion (e.g., nephrotic syndrome)
Immunodeficiency
Immunosuppression
Recent (≤ 12 months) infusion of intravenous immunoglobulin, injection of gamma-globulin, blood/plasma transfusion
Vaccination against measles
Denial of parental consent

**Table 2:** Infant and maternal demographics and measles humoral immune status in the total cohort and the maternal national origin-related subgroups.

	Total Population	Maternal National Origin		
		Greek	Non-Greek	*p*
N	187	136	51	
Infant age [months; median (IQR)]	8.0 (4.0)	8 (3.5)	8 (3.5)	0.235
Male sex [n (%)]	112 (59.9)	80 (58.8)	32 (62.7)	0.738
Maternal age [years; median (IQR)]	31.0 (8.0)	32.0 (8.0)	29.0 (6.0)	0.003
Maternal measles history				0.320
Infection [n (%)]	29 (15.5)	24 (17.6)	5 (9.8)	
Vaccination [n (%)]	110 (58.8)	80 (58.8)	30 (58.8)	
Unknown [n (%)]	48 (25.7)	32 (23.5)	16 (31.4)	
Maternal protective immunity (n = 124)				1.0
Positive [n (%)]	121 (97.6)	82 (97.6)	39 (97.5)	
Equivocal [n (%)]	0	0	0	
Negative [n (%)]	3 (2.4)	2 (2.4)	1 (2.5)	
Maternal antibody levels [mIU/mL, median (IQR)]	1201 (868)	1293 (736)	640 (944)	0.002
Infant protective immunity				0.534
Positive [n (%)]	27 (14.4)	19 (14.0)	8 (15.7)	
Equivocal [n (%)]	7 (3.7)	4 (2.9)	3 (5.9)	
Negative [n (%)]	153 (81.8)	113 (83.1)	40 (78.4)	
Infant antibody levels [mIU/mL, median (IQR)]	56.9 (105)	58.3 (102)	54.2 (124)	0.349

IQR, interquartile range.

**Table 3:** Maternal and infant data in the four infant age-related subgroups.

	Infant Age-Related Subgroups				
	Age 1–4 Months	Age 5–7 Months	Age 8–9 Months	Age 10–12 Months	p *
Infants/mothers’ count	28/21	57/37	49/31	53/35	
Infant age range [months]	1–4	5–7	8–9	10–12 m	
Infant age [months; median (IQR)]	2.0 (2.3)	7.0 (1.0)	8.6 (1.0)	10.5 (1.3)	<0.001
Maternal age [years; median (IQR)]	31.4 (4.8)	31.5 (5.9)	30.0 (5.3)	30.9 (6)	0.509
Maternal history of measles					0.07
Infection [n (%)]	2 (7.1)	15 (26.3)	8 (16.3)	4 (7.5)	
Vaccination [n (%)]	16 (57.1)	27 (47.4)	29 (59.2)	38 (58.8)	
Unknown [n (%)]	10 (35.7)	15 (26.3)	12 (24.5)	11 (20.8)	
Maternal antibody concentrations; [mIU/mL, median (IQR)]	962.8 (791)	1295.0 (823)	921.5 (961)	1285.0 (991)	0.682
Maternal protective immunity (n=121)					0.419
Negative [n (%)]	0	0	1 (3.2)	2 (5.7)	
Equivocal [n (%)]	0	0	0	0	
Positive [n (%)]	21 (100)	37 (100)	30 (96.8)	33 (94.3)	
Infant antibody concentrations [mIU/mL, median (IQR)]	277.7 (758)	87.4 (93)	40 (76)	29.7 (45)	<0.001
Infants’ protective immunity					<0.001
Negative [n (%)]	8 (28.6)	50 (87.7)	45 (91.8)	50 (94.3)	
Equivocal [n (%)]	2 (7.1)	2 (3.5)	2 (4.1)	1 (1.9)	
Positive [n (%)]	18 (64.3)	5 (8.8)	2 (1.1)	2 (1.1)	

* Kruskal–Wallis analysis of variance (ANOVA) or chi square test; IQR, interquartile range.

## Data Availability

The data presented in this study are available on request from the corresponding author.
